# Comprehensive Phytochemical, Antioxidant, and Antibacterial Analysis of *Vitex agnus-castus* L. Essential Oil (VACEO): Insights from ADMET and Molecular Docking Studies

**DOI:** 10.3390/ph18040462

**Published:** 2025-03-25

**Authors:** Dounia Azzouni, Soukaina Alaoui Mrani, Fadoua Bahij, Hind Zejli, Mohammed M. Alanazi, Driss Fadili, Abdelfattah El Moussaoui, Ayman M. Mahmoud, Mustapha Taleb

**Affiliations:** 1 Engineering, Electrochemistry, Modelling and Environment Laboratory, Faculty of Sciences, Sidi Mohamed Ben Abdellah University, Fez 30000, Morocco; soukaina.alaouimrani@usmba.ac.ma (S.A.M.); fadoua.bahij@usmba.ac.ma (F.B.); hind.zejli@usmba.ac.ma (H.Z.); mustapha.taleb@usmba.ac.ma (M.T.); 2 Department of Pharmaceutical Chemistry, College of Pharmacy, King Saud University, Riyadh 11451, Saudi Arabia; mmalanazi@ksu.edu.sa; 3 Chemical Physics, Materials and Environment Laboratory, Faculty of Science and Technology, Moulay Ismaïl University of Meknes, Errachidia 52003, Morocco; driss.fadili@gmail.com; 4 Plant Biotechnology Team, Faculty of Sciences, Abdelmalek Essaadi University, Tetouan 93002, Morocco; a.elmoussaoui@uae.ac.ma; 5 Department of Life Sciences, Faculty of Science and Engineering, Manchester Metropolitan University, Manchester M1 5GD, UK; a.mahmoud@mmu.ac.uk

**Keywords:** *Vitex agnus-castus* L., phytochemical composition, antioxidant activity, antibacterial activity, molecular docking

## Abstract

**Background/Objectives:** The phytochemical composition, antioxidant, and antibacterial properties of *Vitex agnus-castus* L. essential oil (VACEO), extracted from fruit harvested in the Middle Atlas region of Morocco, were investigated. **Methods/Results:** A full chemical analysis of VACEO was performed to identify the principal components of the oil using GC–MS, demonstrating that caryophyllene (13.87%), 1-(4-Isopropylphenyl)-2-methylpropyl acetate (12.20%), and τ-Cadinol (5.41%) were the most important constituents of this oil. In addition, antioxidant activity was evaluated using DPPH, FRAP, TAC, and beta-carotene bleach tests; the oil demonstrated an IC50 of 0.93 ± 0.03 mg/mL (DPPH), an EC50 of 0.146 ± 0.004 mg/mL (FRAP), and a total antioxidant capacity of 0.794 ± 0.02 mg BHT equivalent/g EO, with relative antioxidant activity at 72.69 ± 0.3%. Antibacterial assays revealed inhibition diameters ranging from 13.25 ± 1.00 mm to 21.11 ± 0.25 mm, with minimum inhibitory concentrations (MICs) ranging from 0.02–0.04 mg/mL against *Escherichia coli*, *Staphylococcus aureus*, *Bacillus subtilis*, and *Pseudomonas aeruginosa*. Moreover, ADMET analysis suggested VACEO potential for drug development, with specific insights into pharmacokinetics, safety, and molecular docking, clarifying its interactions with key bacterial proteins. **Conclusions:** These results confirm the promising therapeutic and pharmaceutical potential of VACEO through its bioactive compounds.

## 1. Introduction

Computer-aided drug design (CADD) approaches, an indispensable part of medicinal chemistry in recent years, provide powerful tools for mainstreaming the drug development process into experimental research using computational methods [1,2]. The therapeutic potential of natural plant extracts has been extensively studied, particularly as an alternative treatment to address the limitations of synthetic drugs, such as undesirable side effects and the growing threat of antimicrobial resistance [3]. Essential oils extracted from medicinal plants are rich in bioactive compounds, including terpenes, phenolic compounds, and alkaloids, which are known for their diverse pharmacological activities, such as antioxidant, anti-inflammatory, and antimicrobial effects [4]. Of these medicinal plants, *Vitex agnus-castus* L., commonly known as chaste berry, has attracted particular attention due to its historical use in traditional medicine, notably to treat hormonal imbalances and various reproductive disorders [5]. More recent studies are exploring the potential of VACEO as a potential source of bioactive compounds with wide applications, including antimicrobial and antioxidant agents, which are relevant for therapeutic and preservative purposes [6]. The composition of VACEO has been studied, indicating the presence of a complex blend of bioactive molecules, including monoterpenes, sesquiterpenes, and flavonoids, which contribute to its pharmacological properties. These compounds have demonstrated significant antioxidant activity, which is essential for fighting the oxidative stress associated with chronic disease, as well as antimicrobial properties, which are effective against a variety of bacterial and fungal pathogens. Nevertheless, research remains limited, particularly in terms of identifying regional variations in chemical composition and bioactivity. As essential oils are volatile compounds possessing low molecular weights, allowing for cellular membrane penetration and effective microbial cell targeting, VACEO could be a promising candidate for combating antibiotic-resistant pathogens [7]. However, investigations specifically analyzing the chemical composition and biological activities of VACEO remain relatively limited, in particular, focusing on oils extracted from Moroccan regions, whose bioactivity might be affected by local environmental factors [8]. This study was undertaken to address these gaps by performing a detailed phytochemical analysis of VACEO extracted from Moroccan samples, followed by a comprehensive evaluation of its antioxidant and antibacterial properties [9]. In particular, antioxidant activity has been shown to play a crucial role in preventing oxidative stress, which is implicated in various chronic diseases [10]. VACEO’s potential as an antibacterial agent was also examined against common bacterial pathogens, providing insight into its application in developing natural antimicrobial solutions [11]. Moreover, advanced CADD techniques, including in silico ADMET studies and molecular docking, were employed to assess pharmacokinetic profiles and interactions with bacterial proteins essential for pathogen survival and resistance [12,13,14]. These advanced computational methods allow the pharmacokinetic profiles of VACEO to be assessed, providing insight into its potential drug-like nature and its interactions with bacterial proteins that are essential for pathogen survival and resistance [15]. These kinds of investigations help to streamline the drug development process by predicting bioavailability, toxicity, and target interaction profiles prior to clinical trials [16]. These methods provide a comprehensive assessment of the potential of VACEO as a multifunctional natural product with applications in medicine, particularly as a source of natural antioxidants and antimicrobials. These findings contribute to the larger field of bioactive compounds of plant origin and support the exploration of sustainable natural resources for therapeutic development.

## 2. Results and Discussion

### 2.1. Essential Oils’ Chemical Composition

**Figure 1:**
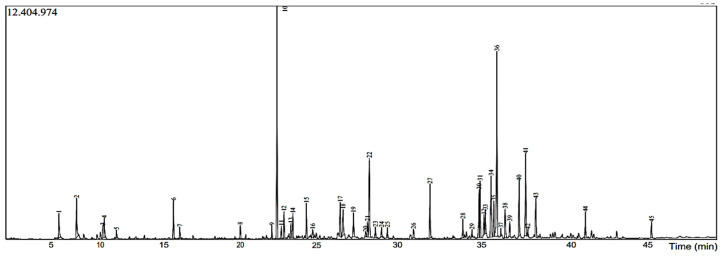
Chromatogram of VACEO.

Distillation extracts about 5.4% of VACEO, meaning 100 g of plant fruit provides 5.4 g of oil. The yield depends on factors such as harvest conditions, climate, and soil type but is generally favorable. The phytochemical analysis identified its main compounds (Figure 1 and Table 1), including Caryophyllene (13.87%), 1-(4-Isopropylphenyl)-2-methylpropyl acetate (12.20%), and τ-Cadinol (5.41%). Their chemical structures are presented in Table 2.

### 2.2. Antioxidant Activities

The antioxidant properties (Table 3) of VACEO were assessed and compared with the standard antioxidants BHT, ascorbic acid, and quercetin. The DPPH assay showed an IC50 of 0.93 ± 0.03 for VACEO, indicating moderate antioxidant activity but weaker than BHT (0.11 ± 0.001 mg/mL).

In the FRAP assay, VACEO had an EC_50_ of 0.146 ± 0.004, again showing good but lesser activity compared to quercetin (0.03 ± 0.004 mg/mL). The relative antioxidant activity of VACEO was 72.69 ± 0.3%, compared to ascorbic acid set at 100%, highlighting its substantial, though not maximal, effectiveness. Additionally, the total antioxidant capacity (TAC) of VACEO was 0.794 ± 0.02 mg Eqv BHT/g EO. Overall, VACEO demonstrates significant antioxidant potential, though it is not as potent as some standard antioxidants. This result was explained by the richness of our VACEO with compounds having an excellent antioxidant potential. Golkar and Mottar [17] were also able to conclude that VACEO has antioxidant activity and is rich in bioactive compounds. Our results are in line with other previous research carried out on the same plants but in different countries, such as Nigeria, China, and Switzerland [18,19,20].

### 2.3. Antibacterial Activity

The antibacterial activity of VACEO was evaluated against four bacterial strains: *Escherichia coli*, *Staphylococcus aureus*, *Bacillus subtilis*, and *Pseudomonas aeruginosa* (Table 4). In comparison with the standard antibiotic kanamycin, VACEO exhibited notable inhibitory effects. For *E. coli*, VACEO produced an inhibition diameter (ID) of 18.25 ± 0.75 mm, slightly lower than kanamycin’s 19.3 ± 1.56 mm, with a minimum inhibitory concentration (MIC) of 0.02 mg/mL, higher than kanamycin’s 0.002 mg/mL. *Staphylococcus aureus* showed comparable results, with VACEO achieving an ID of 21.11 ± 0.25 mm and a MIC of 0.02 mg/mL, similar to kanamycin’s performance.

However, against *Bacillus subtilis*, VACEO exhibited a smaller ID of 13.25 ± 1.00 mm and a higher MIC of 0.04 mg/mL compared to kanamycin. Similarly, for *Pseudomonas aeruginosa*, VACEO demonstrated an ID of 17.35 ± 1.00 mm and a MIC of 0.02 mg/mL, again higher than kanamycin’s MIC of 0.004 mg/mL. In other studies, VACEO has shown antibacterial efficacy against several strains, including *S. aureus*, while an Iranian study showed resistance to *Listeria monocytogenea* [21,22]. Overall, while VACEO displayed significant antibacterial activity across all tested strains, its effectiveness varied, being generally less potent than kanamycin but still showing promising inhibitory effects.

### 2.4. ADMET Study

**Figure 2:**
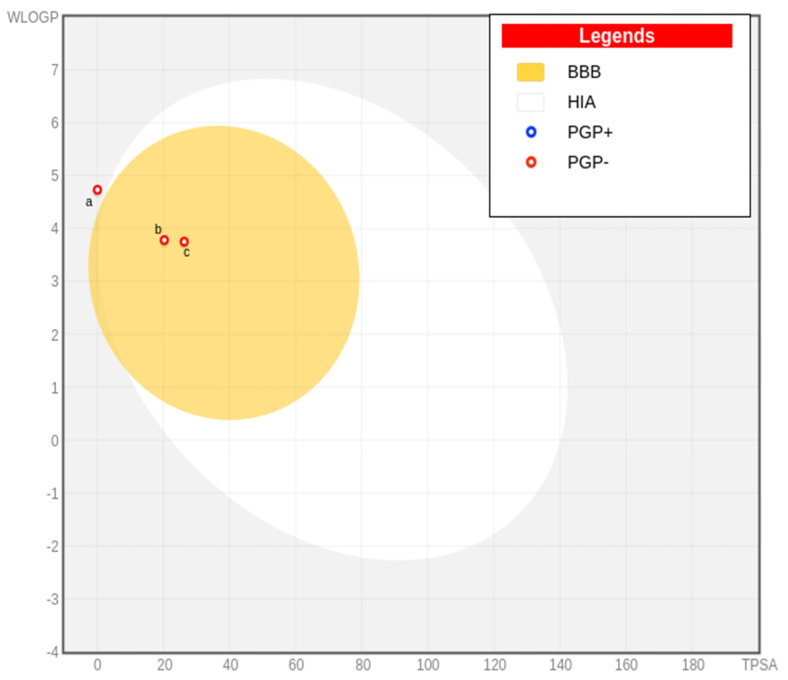
The egg-boiled model illustrates the three major constituents of VACEO: **a**—Caryophyllene, **b**—1-(4-Isopropylphenyl)-2-methylpropyl acetate, and **c**—τ-Cadinol. The model highlights their structural characteristics and predicts pharmacokinetic properties. Abbreviations: BBB—Blood–Brain Barrier permeability, HIA—Human Intestinal Absorption, PGP^+^: Positive substrate for P-glycoprotein (indicating potential efflux), and PGP^−^: Negative substrate for P-glycoprotein (indicating no interaction).

**Figure 3:**
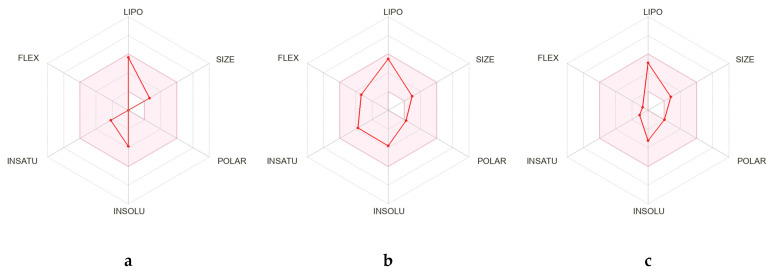
The bioavailability radar plot for the three major constituents of VACEO: **a**—Caryophyllene, **b**—1-(4-Isopropylphenyl)-2-methylpropyl acetate, and **c**—τ-Cadinol. The plot visualizes key pharmacokinetic parameters, including Lipophilicity (LIPO), Size (SIZE), Polarity (POLAR), Insolubility in water (INSOLU), Flexibility (FLEX), and the degree of Insaturation of the molecule (INSATU), which collectively provide a comprehensive overview of the bioavailability of each compound.

The three oil-extracted molecules were evaluated using the SwissADME online tool based on various pharmacokinetic and physicochemical properties, including their compliance with drug-likeness rules like Lipinski’s (L), Ghose’s (G), Veber’s (V), Egan’s (E), and Muegge’s (M). All those properties are shown in Table 5. Molecule a violated Lipinski’s rule due to the absence of hydrogen acceptors or donors [23], while molecules b and c met the criteria. Molecules b and c were identified as not being Pgp substrates, which is advantageous as Pgp (P-glycoprotein) can reduce drug efficacy by expelling drugs from cells [24]. The egg-boiled model analysis in Figure 2 indicated that both b and c were also found to be BBB permeants, indicating their ability to cross the blood–brain barrier (BBB), a key factor for central nervous system (CNS) drug candidates [25]. The bioavailability radars of the three molecules were obtained and are shown in Figure 3. The bioavailability radar showed that all properties of the three molecules are within the colored area, suggesting their drug-like characteristics. Additionally, b and c showed high gastrointestinal (GI) absorption, enhancing their potential for oral delivery. All molecules had a moderate bioavailability score (BS) of 0.55, reflecting their general drug-like nature. Regarding PAINS alerts (PA) and Brenk alerts (BA), none of the molecules raised concerns, suggesting a low likelihood of promiscuous activity and toxicity. Lastly, molecule a showed leadlikeness violations (LV), which may affect its suitability as a drug lead. In contrast, molecules b and c did not exhibit such violations, making them more promising candidates for further development.

A pharmacokinetic and drug-likeness analysis of the compounds under study was performed. The parameters include the following: MW: molecular weight, influencing absorption and permeability. RB: number of rotatable bonds, indicating molecular flexibility. HA: number of hydrogen bond acceptors, affecting solubility and binding. HB: number of hydrogen bond donors, impacting interactions with biological targets. MR: molar refractivity, reflecting steric and electronic properties. MLOGP: predicted log partition coefficient (logP), a measure of lipophilicity. Lipinski (L): rule of five violations. Ghose (G): Ghose filter violations. Veber (V): Veber’s criteria violations. Egan (E): Egan’s filter violations. Muegge (M): Muegge’s criteria violations. Pgp substrate (Pgp): indicates if the compound is a substrate for P-glycoprotein. BBB: blood–brain barrier permeability. GI absorption: gastrointestinal absorption. Bioavailability Score (BS): a prediction of oral bioavailability. PAINS alerts (PA): pan-assay interference alerts. Brenk alerts (BA): structural alerts based on undesirable motifs. Leadlikeness violations (LV): molecular property assessments for lead compounds. CYP2C19 (C19) and CYP2C9 (C9): interaction predictions with cytochrome P450 enzymes, important for drug metabolism.

**Figure 4:**
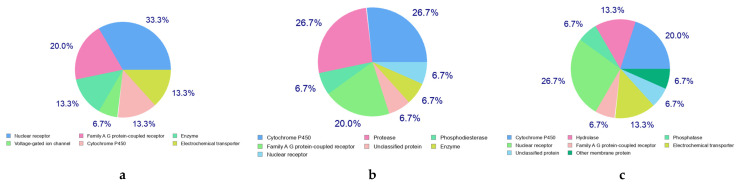
The predicted target for the three major constituents of VACEO: **a**—Caryophyllene, **b**—1-(4-Isopropylphenyl)-2-methylpropyl acetate, and **c**—τ-Cadinol. The figure illustrates the predicted biological targets of each compound, providing insights into their potential pharmacological activities and interactions. Percentages are rounded to one decimal place, which may result in a total slightly exceeding 100%. Images are generated using the Swissadme web app.

The predicted target profiles for the studied molecules depicted in Figure 4, based on data obtained from the SwissTargetPrediction website, include interactions with several important protein families and enzymes, such as Nuclear Receptors, Family A G Protein-Coupled Receptors (GPCRs), Cytochrome P450 Enzymes, Hydrolases, Phosphatases, and Electrochemical Transporters.

The predicted toxicity of the studied molecules was evaluated based on their lethal dose for 50% of the population (LD50) values, toxicity class, average similarity, and prediction accuracy using the Protox-3.0 web server platform [26]. The results presented in Table 6 suggest that all three molecules are predicted to have low toxicity, with molecule **c** being somewhat more toxic than **a** and **b**, with a significantly lower predicted LD50 of 2830 mg/kg. All molecules have an average similarity higher than 82% and a prediction accuracy higher than 70%.

**Figure 5:**
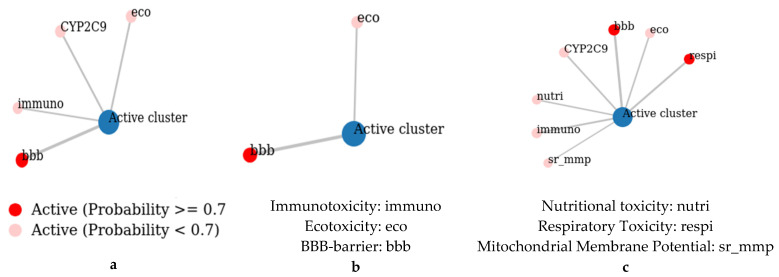
Toxicity profile of the studied compound using the ProTox-3.0 server. With **a**—Caryophyllene, **b**—1-(4-Isopropylphenyl)-2-methylpropyl acetate, and **c**—τ-Cadinol.

The predicted toxicity activities of the studied molecules, depicted in Figure 5, reveal various risks associated with the use of the oil-extracted molecules. Firstly, **a** is predicted to be Immunotoxicity Active with a probability of 0.54, meaning it may trigger immune responses. It is also BBB-barrier Active (0.97), indicating a strong potential to cross the blood–brain barrier, which could pose neurological risks. Additionally, it shows Ecotoxicity Activity (0.68), suggesting environmental hazards, and Cytochrome CYP2C9 Activity (0.67), implying that it may interfere with this key metabolic enzyme. Secondly, **b** is primarily BBB-barrier Active (0.96), indicating its potential to cross the blood–brain barrier. It also has an Ecotoxicity Activity (0.58), suggesting possible environmental impact. Lastly, **c** exhibits a broader range of toxic activities. It is Respiratory Toxicity Active (0.75), posing risks to the respiratory system, and Immunotoxicity Active (0.69). Like the other molecules, it is BBB-barrier Active (0.88) and Ecotoxicity Active (0.60). Additionally, it is Nutritional Toxicity Active (0.56), indicating potential adverse effects on nutritional health, and Mitochondrial Membrane Potential (MMP) Active (0.50), suggesting it may alter cellular energy production [27]. It also shows activity against Cytochrome CYP2C9 (0.66), which could lead to metabolic complications.

### 2.5. Molecular Docking

Table 7 shows the binding energy affinities between the three extracted oil molecules and various protein targets. Also, the interaction of those proteins and the original ligand. Each protein is associated with specific bacterial functions. These targets include GyrA and GyrB (involved in DNA replication in *Escherichia coli*), Topoisomerase IV (another crucial enzyme in *E. coli*), Beta-lactamase (a resistance enzyme in *Staphylococcus aureus*), Penicillin-binding protein 2a (PBP2a) (associated with methicillin resistance in *S. aureus*), Sortase A (a virulence factor in *S. aureus*), Staphylocoagulase (involved in immune evasion by *S. aureus*), DNA polymerase III (essential for DNA replication in *E. coli*), MurA (a key enzyme in bacterial cell wall biosynthesis in *E. coli*), LasR (a quorum-sensing regulator in *Pseudomonas aeruginosa*), and Elastase (a tissue-degrading enzyme in *P. aeruginosa*). These binding affinities offer insights into how well the oils might inhibit these proteins, potentially leading to antibacterial effects.

The binding affinity trends presented in Table 7 highlight several key insights. For GyrA (PDB: 4Z2C), the original ligand exhibits the strongest binding affinity, indicating that it likely fits well within the active site and forms more stable interactions compared to the other ligands. On the other hand, Ligand a shows the weakest binding affinity, suggesting it may not interact as effectively with GyrA. In the case of GyrB (PDB: 5L3J), Ligand b surpasses the original ligand in binding affinity, making it a promising candidate as a potent inhibitor of GyrB. This suggests that Ligand b could serve as a more effective alternative to the original ligand for targeting this protein.

For Topoisomerase IV (PDB: 1S14), the original ligand demonstrates the highest binding affinity, outperforming the other ligands, which show notably lower affinities. This indicates that the original ligand remains the most effective for inhibiting Topoisomerase IV, maintaining its position as the preferred candidate.

When examining MurA (PDB: 1YBG), the original ligand again demonstrates the strongest binding affinity, reinforcing its potential as an effective inhibitor. This strong interaction suggests that the original ligand is well-suited for targeting MurA. Interestingly, with Sortase A (PDB: 6R1V), Ligand c exhibits a higher binding affinity than the original ligand, which could indicate a better fit or stronger interactions within the Sortase A binding site. This finding suggests that Ligand c could be a promising alternative for further exploration.

In terms of relative strengths of ligands, Ligand b shows strong performance across multiple targets, particularly in the cases of GyrB and LasR, highlighting its potential as a broad-spectrum inhibitor. Conversely, Ligand a consistently shows lower binding affinities compared to the original ligands, indicating it may be less effective overall. On the other hand, Ligand c demonstrates competitive binding, especially with Sortase A and DNA polymerase III, making it worth further investigation for its potential inhibitory properties.

**Figure 6:**
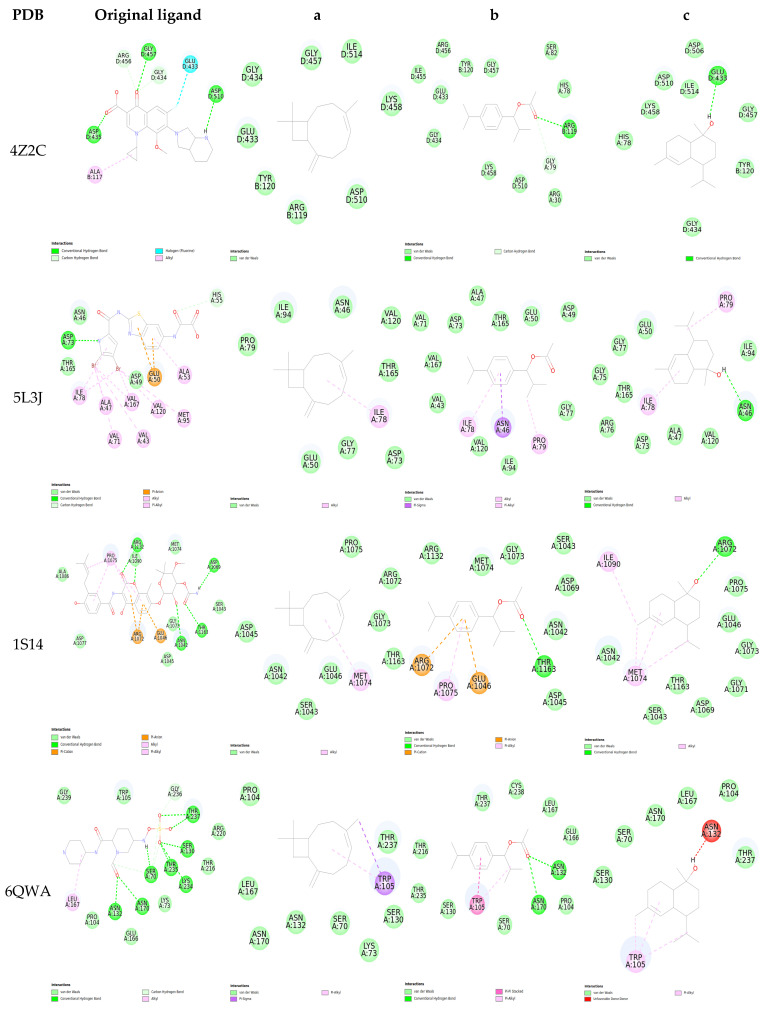

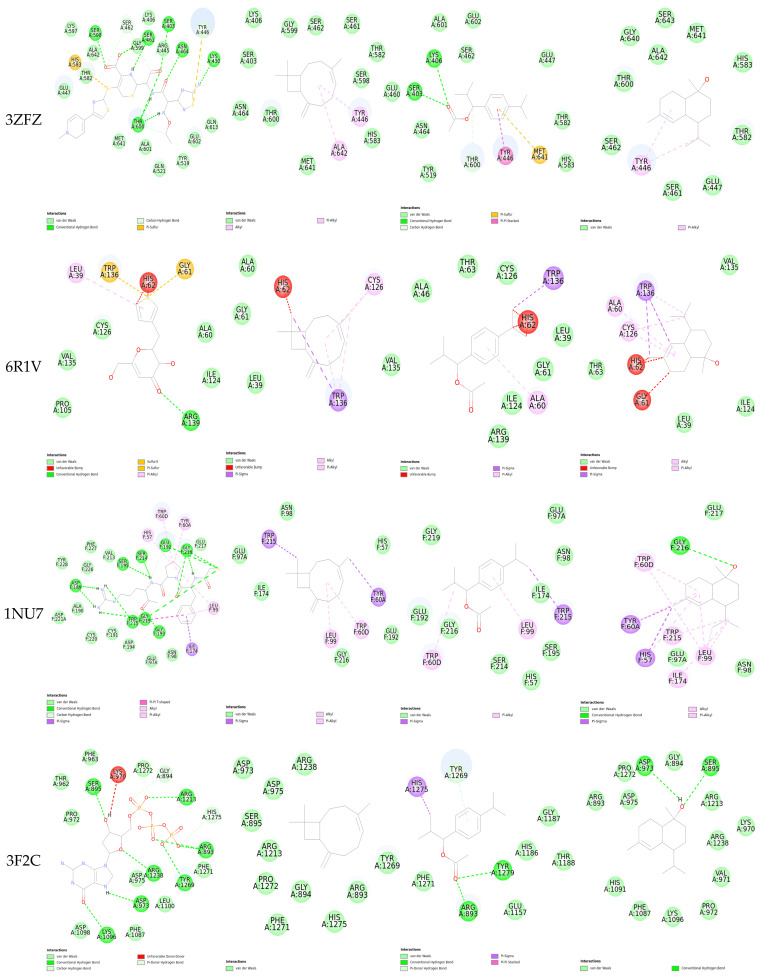

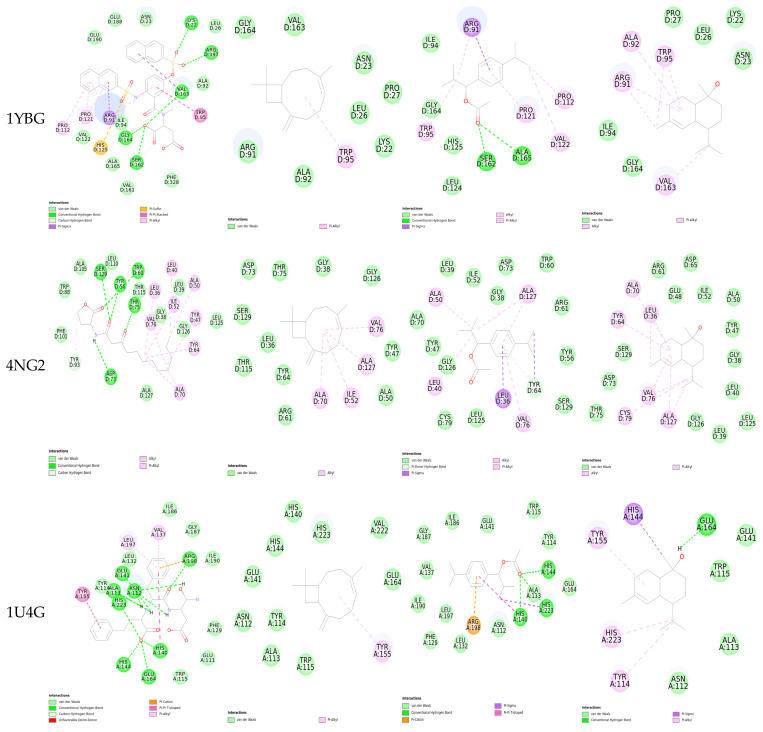
Two-dimensional visualization of the interaction between ligands and protein residues. With **a**—Caryophyllene, **b**—1-(4-Isopropylphenyl)-2-methylpropyl acetate, and **c**—τ-Cadinol.

**Figure 7:**
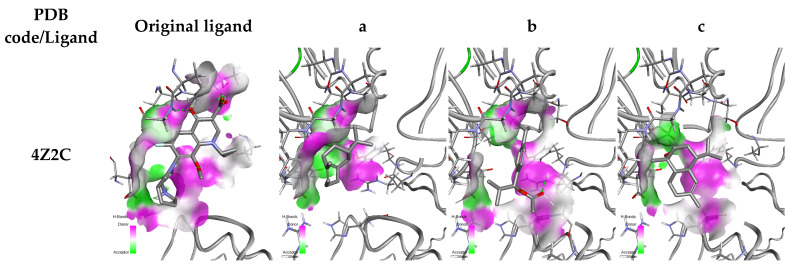

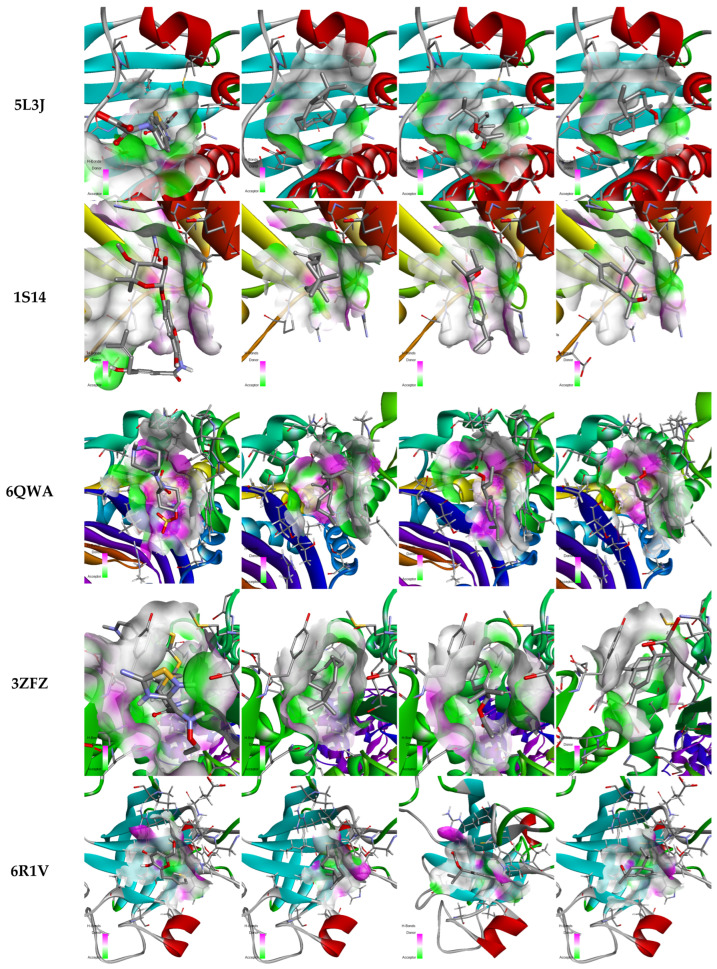

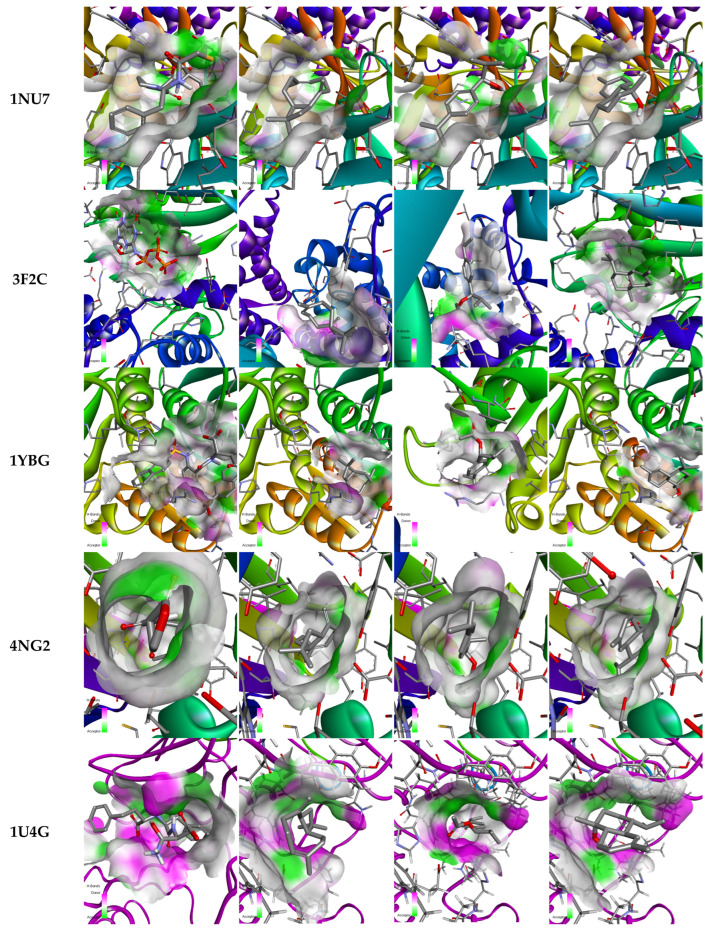
Three-dimensional visualization of the interaction between ligands and protein residues. With **a**—Caryophyllene, **b**—1-(4-Isopropylphenyl)-2-methylpropyl acetate, and **c**—τ-Cadinol.

The 2D interactions between the ligands and the selected protein targets reveal important insights into their binding modes (Figure 6 and Figure 7), particularly focusing on the hydrogen bonding patterns that play a crucial role in stabilizing these interactions. For GyrA (PDB: 4Z2C), the original ligand exhibits strong hydrogen bonding with ASP D: 435, GLY D: 457, and ASP D: 510, suggesting a highly stable interaction within the active site. In contrast, Ligand b interacts with ARG B: 119, while Ligand c forms a bond with GLU D: 433. Ligand a, however, lacks hydrogen bonding with all targets due to the absence of a hydrogen acceptor or donor; this results in a less stable interaction.

In GyrB (PDB: 5L3J), the original ligand’s interaction with ASP A: 73 suggests a strong and stable binding. However, Ligand b does not form any hydrogen bonds, indicating potentially less effective binding. Ligand c interacts with ASN A: 46, which may offer an alternative binding mode, though possibly not as strong as the original ligand.

For Topoisomerase IV (PDB: 1S14), the original ligand forms hydrogen bonds with ARG A: 1132, ASN A: 1042, and THR A: 1163, reflecting a robust interaction. Ligand b shares a bond with THR A: 1163, suggesting some similarity in binding mode with the original ligand, while Ligand c binds to ARG A: 1072, indicating a different interaction pattern. The Beta-lactamase (PDB: 6QWA) original ligand forms a complex network of hydrogen bonds with several residues, including ASN A: 132, ASN A: 170, SER A: 70, and THR A: 237, indicating a very stable binding. Ligand b shows interactions with ASN A: 132 and ASN A: 170, suggesting it might mimic some of the original ligand’s binding characteristics, while Ligand c also interacts with ASN A: 132 and ASN A: 170, potentially indicating competitive binding.

In Penicillin-binding protein 2a (PBP2a, PDB: 3ZFZ), the original ligand’s interactions with residues such as SER A: 598, ASN A: 464, and THR A: 600 highlight its strong binding affinity. Ligand b forms bonds with LYS A: 407 and SER A: 403, suggesting a potential for effective binding, though not as robust as the original ligand. Ligand c lacks hydrogen bonding, which may indicate weaker interactions.

Sortase A (PDB: 6R1V) interactions are limited, with only the original ligand showing a hydrogen bond with ARG A:139, suggesting it may have a more stable interaction compared to Ligands b and c, which do not form any hydrogen bonds.

For Staphylocoagulase (PDB: 1NU7), the original ligand’s extensive hydrogen bonding network, including interactions with ASP F: 189, SER F: 195, and GLY F: 216, points to a strong and stable binding. Ligand c forms a bond with GLY F: 216, indicating some level of effective binding, while Ligand b shows no hydrogen bonding, potentially leading to weaker interactions.

In DNA polymerase III (PDB: 3F2C), the original ligand’s hydrogen bonds with residues like SER A: 895 and ARG A: 1213 highlight its strong binding affinity. Ligand b shares a bond with ARG A: 893 and TYR A: 1279, indicating a somewhat similar binding mode, while Ligand c interacts with SER A: 895 and ASP A: 973, suggesting competitive binding.

For MurA (PDB: 1YBG), the original ligand forms hydrogen bonds with LYS D: 22, ARG D: 397, and SER D: 162, indicating a stable interaction. Ligand b also interacts with SER D: 162 and ALA D: 165, suggesting some binding potential, while Ligand c does not form hydrogen bonds, indicating weaker interactions.

Lastly, in LasR (PDB: 4NG2), the original ligand forms hydrogen bonds with SER D: 129, TYR D: 56, and THR D: 75, which likely contribute to strong binding. The absence of hydrogen bonding for Ligands a, b, and c suggests they may not interact as effectively with the target. Similarly, in Elastase (PDB: 1U4G), the original ligand’s interactions with HIS A: 223 and GLU A: 141 indicate strong binding, while Ligand b interacts with HIS A: 144 and HIS A: 223, and Ligand c binds to GLU A: 164, suggesting some potential for competitive binding.

## 3. Material and Methods

### 3.1. Plant Material

*V. agnus-castus* fruits were collected from an area called Khenifra (Latitude: 32°56′05″ North; Longitude: 5°39′42″ West; altitude: 827 m) (Middle Atlas, Morocco) in June–October 2023 (blooming period) with 2298/4-16-2/Kh as reference code.

### 3.2. Extract Preparation

The fruit of *V. agnus-castus* was washed and dried under shade before extraction and then hydrodistilled at a ratio of 1/10 solid/liquid using a Clevenger instrument lasting 3 h, in line with the European Pharmacopoeia [28]. The obtained essential oil was preserved in a 4 °C flask before use.

### 3.3. Phytochemical Analysis

The essential oil’s chemical compositions were analyzed using a Hewlett-Packard apparatus, which was fitted with an apolar capillary column (RTxi-5 Sil MS) (30 m × 0.25 mm ID, 0.25 µm film thickness), temperature set from 50 to 250 °C at 5 °C/min, and connected to a mass spectrometer (HP 5973). Helium was utilized as the vector gas in fractionated mode, with a flow rate of 112 µL/min and a ratio of 1/74.7. By verifying their identities using MS (NIST98 spectra collection), the components were identified. A 1 μL sample was manually injected (1/50 in hexane) [29,30].

### 3.4. Antioxidant Activity

#### 3.4.1. 2,2-Diphenylpicrylhydrazyl Method (DPPH)

The DPPH method involved adding 100 µL of essential oil to 750 µL of a methanolic solution of DPPH (0.004%) [31]. After 30 min of incubation at room temperature, the absorbance was measured at 517 nm. The percentage of DPPH inhibition was calculated using the equation:

$$PI(\%)=(\frac{A_{0}-A}{A_{0}})\times 100$$

where:

*PI* is the percentage of inhibition;

*A*_0_ is the absorbance of the DPPH of negative control (NC);

*A* is the absorbance of DPPH of the sample;

The IC_50_ values were obtained from the inhibition percentage graph against extract concentration.

#### 3.4.2. Ferric Reducing Antioxidant Power (FRAP)

For the FRAP test, 500 µL of phosphate buffer (0.2 M, pH = 6.6), 500 µL of potassium ferricyanide (1%), and 100 µL of different sample concentrations dissolved in methanol were combined. After incubating at 50 °C for 20 min, 500 µL of aqueous TCA solution (10%), 500 µL of distilled water, and 100 µL of 0.1% FeCl_3_ were added. Absorbance was measured at 700 nm, and results were expressed as 50% effective concentration (EC_50_) [32].

#### 3.4.3. Total Antioxidant Capacity Test (TAC)

For the TAC test, 25 µL of the extract was mixed with a reagent solution containing 0.6 M sulfuric acid (H_2_SO_4_), 28 mM sodium phosphate (Na_2_HPO_4_), and 4 mM ammonium molybdate ((NH_4_)_2_MoO_4_). The mixture was then incubated at 95 °C for 90 min. After incubation, the absorbance was measured at 695 nm using a spectrophotometer. The total antioxidant capacity was determined by comparing the absorbance of the sample with a standard curve of BHT. The results were expressed as micrograms of BHT equivalent per gram of extract (mg BHT/g dry weight), providing an assessment of the extract’s antioxidant potential based on the behavior of ascorbic acid under the same conditions [33].

#### 3.4.4. Beta-Carotene Bleaching Inhibition Assay

The Beta-Carotene Bleaching Inhibition Test evaluates antioxidant activity by measuring the ability to prevent beta-carotene bleaching in a beta-carotene/linoleic acid model [34]. Linoleic acid, Tween 80, and a beta-carotene solution are mixed, with chloroform removed via rotary evaporation. Hydrogen peroxide is added to form an emulsion, which is then combined with the test sample or ascorbic acid (reference antioxidant), and a control with methanol. Absorbance at 470 nm is measured initially and at intervals up to 120 min in a 50 °C water bath to assess the inhibition of beta-carotene bleaching. The antioxidant activity was assessed based on the inhibition of beta-carotene bleaching, calculated using the formula:

$$AA(\%)=(\frac{A_{E}}{A_{PC}})\times 100$$

where:

*AA* % is the percentage of antioxidant activity;

*A_E_* is the absorbance after 120 min with the sample;

*A_PC_* is the absorbance after 120 min of the positive control.

### 3.5. Antibacterial Activity

The Antibacterial efficacy was first assessed using the disk diffusion method [35] to identify the antimicrobial potency of our essential oil. The samples were tested against *Escherichia coli* (ATCC 25922), *Staphylococcus aureus* (ATCC 29213), *Bacillus subtilis* (ATCC 6633), and *Pseudomonas aeruginosa* (ATCC 27853). To determine the minimum inhibitory concentration (MIC), our sample was then micro-diluted in 96-well microplates, following the protocol described by [36].

### 3.6. ADMET Investigation

To predict the absorption, distribution, metabolism, and excretion (ADME) pharmacokinetics, along with the physicochemical properties of the compound under investigation, we utilized the online Swiss ADME tool (http://swissadme.ch/, accessed 27 July 2024) [37]. Drug-likeness was assessed using Lipinski’s Rule of Five, which considers a molecular weight below 500 g/mol, fewer than five hydrogen bond donors, no more than ten hydrogen bond acceptors, a partition coefficient (Log P) less than 5, and allowance for only one rule violation. Additionally, molecular target predictions were performed using the web tool (swisstargetprediction.ch, accessed on 27 July 2024). The toxicity of all compounds was calculated using the ProTox-3.0 server [38], a web server that integrates 33 prediction models (accessed on 27 July 2024).

### 3.7. Docking Study

To explore the interactions between the selected ligands and the active site associated with the selected proteins, molecular docking modelling was utilized. The target proteins and their original ligand were retrieved from the RCSB Protein Data Bank. The active site was identified based on the original ligand’s location within the protein structure. The target proteins were prepared by removing the original ligand, water molecules, and other non-protein components, except for the essential ligands involved in the target metabolism. A grid box was created based on the position of the original ligand. AutoVina version 1.2.3 was then used to analyze the interactions between the ligands and the proteins [26]. The ligand poses within the target protein were visualized by the Biovia visualization tools 2024.

## 4. Conclusions

As a potential natural therapeutic agent, VACEO exhibits both antioxidant and antibacterial properties. The phytochemical analysis demonstrated a complex mixture of bioactive compounds, including caryophyllene and 1-(4-Isopropylphenyl)-2-methylpropyl acetate, which contribute to the oil’s biological efficacy. Their antioxidant activity, observed in numerous tests, indicates that VACEO has substantial free-radical binding potential, though slightly lower than that of standard antioxidants. Moreover, the antibacterial tests highlighted its inhibitory effects on a range of bacterial strains, underlining its suitability as a natural antimicrobial agent. Both ADMET predictions and molecular docking analyses confirm the suitability of VACEO for drug development, with favorable pharmacokinetic properties and specific interactions with key bacterial proteins, especially those involved in resistance mechanisms. As a result, VACEO represents a strong potential candidate as a natural therapeutic agent for further development, with applications extending to antioxidant and antibacterial formulations. Future studies should explore in vivo efficiency and safety to facilitate potential pharmaceutical applications and expand understanding of the VACEO bioactive profile.

## Figures and Tables

**Table 1:** Phytochemical constituents of VACEO.

Peak	Retention Time	Phytochemical Compounds	Area (%)
**1**	7.899	2-Pinene	1.61
**2**	9.028	Bicyclo [3.1.0]hexane, 4-methylene-1-(1-methylethyl)	2.67
**3**	10.710	D-Limonene	1.36
**4**	10.799	Eucalyptol	1.24
**5**	11.583	Gamma.-Terpinene	0.42
**6**	15.228	Terpinen-4-ol	2.37
**7**	15.634	(1S)-1,3,3-trimethylnorbornan-2-ol	0.67
**8**	19.513	Bicycloelemene	0.72
**9**	21.531	Alpha.-Gurjunene	0.69
**10**	21.860	Caryophyllene	13.87
**11**	22.136	Alpha.-Bergamotene	0.63
**12**	22.310	Cyclohexene, 3-(1,5-dimethyl-4-hexenyl)-6	1.41
**13**	22.752	alpha.-Humulene	0.83
**14**	22.872	Alloaromadendrene	1.43
**15**	23.743	(1S,2E,6E,10R)-3,7,11,11-Tetramethylbicyclo[8.1.0]undeca-2,6-diene	2.01
**16**	24.150	4-Isopropyl-1,6-dimethyl-1,2,3,4,4a,7,8,8a-octahydro-1-naphthalenol	0.63
**17**	25.903	1H-Cycloprop[e]azulen-7-ol, decahydro-1,1,7-trimethyl-4-methylene-, [1ar (1a.alpha.,4a.alpha.,7.beta.,7a.beta.,7b.alpha.)]	2.39
**18**	26.086	5-Oxatricyclo[8.2.0.04,6]dodecane, 4,12,12-trimethyl-9-methylene-, (1R,4R,6R,10S)	2.17
**19**	26.757	Ledol	1.63
**20**	27.525	Isospathulenol	0.42
**21**	27.657	10,10-Dimethyl-2,6-dimethylenebicyclo[7.2.0]undecan-5.beta.-ol	0.99
**22**	27.773	Tau.-Cadinol	5.41
**23**	28.160	Viridifloro	0.69
**24**	28.553	Isoaromadendrene epoxide	0.60
**25**	28.928	alpha.-Bisabolol	0.60
**26**	30.602	2,5-Dimethylbicyclo[3.3.0]oct-6-en-8-one	0.52
**27**	31.653	Androsta-4,6-dien-3-one, 17-.beta.-hydroxy	3.38
**28**	33.761	(E)-4-(1,3,3-trimethylnorcaran-2-yl)but-3-en-2-one	1.09
**29**	34.342	Kolavelool	0.46
**30**	34.802	cis-Valerenyl acetate	3.47
**31**	34.853	1,1,2,3,3,5-hexamethyl-2H-indene	3.74
**32**	35.107	3-Buten-2-one, 3-methyl-4-(2,6,6-trimethyl-1-cyclohexen-1-yl)-	1.13
**33**	35.199	9,19-Cycloergost-24(28)-en-3-ol, 4,14-dimethyl-, acetate, (3.beta.,4.alpha.,5.alpha.)	1.22
**34**	35.566	(3E,6E)-5-isopropylidene-6-methyl-deca-3,6,9-trien-2-one	5.23
**35**	35.735	Humulane-1,6-dien-3-ol	2.22
**36**	35.934	1-(4-Isopropylphenyl)-2-methylpropyl acetate	12.20
**37**	36.190	1H-Naphtho[2,1-b]pyran, 3-ethenyldodecahydro-3,4a,7,7,10a-pentamethyl-, [3R-(3.alpha.,4a.beta.,6a.alpha.,10a.beta.,10b.alpha.)]	0.40
**38**	36.462	Seychellene	2.11
**39**	36.761	Kaur-15-ene	0.86
**40**	37.358	Podocarpa-8,11,13-triene, 13-isopropyl	3.58
**41**	37.777	cis-3,14-Clerodadien-13-ol	5.36
**42**	37.915	Androsta-1,4-dien-3-one, 6.beta.,17.beta.-dihydroxy-, 17-acetate	0.45
**43**	38.427	Kolavelool	2.65
**44**	41.603	(1R,2R,4aS,8aS)-1-(2-(Furan-3-yl)ethyl)-2,5	1.56
**45**	45.832	Rotundifuran	0.90
		Total (%)	99.98

**Table 2:** Major constituents of VACEO.

	Name	Chemical Formula	Structure	Molar Mass (g/mol)
**a**	Caryophyllene	C_15_H_24_	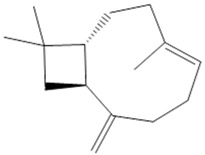	204.35
**b**	1-(4-Isopropylphenyl)-2-methylpropyl acetate	C_15_H_22_O_2_	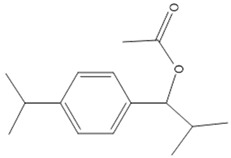	234.34
**c**	τ-Cadinol	C_15_H_26_O	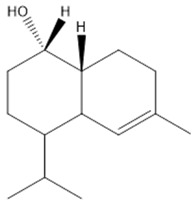	222.36

**Table 3:** Assessment of antioxidant properties of VACEO.

	VACEO	BHT	Acid Ascorbic	Quercetin
DPPH (IC _50_ mg/mL)	0.93 ± 0.03	0.11 ± 0.001	-	-
FRAP (EC_50_ mg/mL)	0.146 ± 0.004	-	-	0.03 ± 0.004
Relative antioxidant activity in %	72.69 ± 0.3%	-	100%	-
TAC in mg eqv BHT/g BHT/g EO	0.794 ± 0.02	-	-	-

**Table 4:** Evaluation of antibacterial activity of VACEO.

	*Escherichia coli*		*Staphylococcus aureus*		*Bacillus subtilis*		*Pseudomonas aeruginosa*	
	ID (mm)	MIC (mg/mL)	ID (mm)	MIC (mg/mL)	ID (mm)	MIC (mg/mL)	ID (mm)	MIC (mg/mL)
VACEO	18.25 ± 0.75	0.02	21.11 ± 0.25	0.02	13.25 ± 1.00	0.04	17.35 ± 1.00	0.02
Kanamycin	19.3 ± 1.56	0.002	21.4 ± 1.2	0.016	19.3 ± 1.5	0.004	17.00 ± 0.00	0.004

**Table 5:** Selected physicochemical parameters of three molecules, **a**—Caryophyllene, **b**—1-(4-Isopropylphenyl)-2-methylpropyl acetate, and **c**—τ-Cadinol.

Mol	MW	RB	HA	HB	MR	MLOGP	GI	BBB	Pgp	C19	C9	L	G	V	E	M	BS	PA	BA	LV
**a**	204.35	0	0	0	68.78	4.63	Low	No	No	Yes	Yes	1	0	0	0	1	0.55	0	1	2
**b**	234.33	5	2	0	71.31	3.65	High	Yes	No	No	No	0	0	0	0	0	0.55	0	0	2
**c**	222.37	1	1	1	70.72	3.67	High	Yes	No	Yes	No	0	0	0	0	1	0.55	0	1	1

**Table 6:** Predicted toxicity results obtained from the ProTox-3.0 web platform [26]. With **a**—Caryophyllene, **b**—1-(4-Isopropylphenyl)-2-methylpropyl acetate, and **c**—τ-Cadinol. The figure presents the predicted lethal dose for 50% of the population (LD50) for each compound, along with the class (toxicity classification), average similarity (similarity to known toxic compounds), and prediction accuracy (confidence of the toxicity prediction), offering insights into their potential toxicity profiles.

	Predicted LD50: mg/kg	Class	Average Similarity	Prediction Accuracy
**a**	5300	5	86.96%	70.97%
**b**	5000	5	82.83%	70.97%
**c**	2830	5	96.55%	72.90%

**Table 7:** Affinities of the most stable poses in kcal/mol. With **a**—Caryophyllene, **b**—1-(4-Isopropylphenyl)-2-methylpropyl acetate, and **c**—τ-Cadinol.

Protein/Ligand	PDB Code	Original Ligand	a	b	c
GyrA	4Z2C	−5.79	−4.59	−4.424	−5.007
GyrB	5L3J	−6.589	−5.923	−6.915	−5.812
Topoisomérase IV	1S14	−7.367	−5.967	−5.572	−5.483
Beta-lactamase	6QWA	−7.618	−6.279	−7.083	−6.436
Penicillin-binding protein 2a (PBP2a)	3ZFZ	−8.985	−6.96	−7.069	−6.656
Sortase A	6R1V	−4.613	−5.053	−4.815	−5.366
Staphylocoagulase	1NU7	−8.955	−8.014	−6.296	−7.074
DNA polymerase III	3F2C	−8.311	−5.267	−5.446	−6.063
MurA	1YBG	−10.27	−6.56	−6.667	−6.282
LasR	4NG2	−9.611	−7.405	−8.762	−8.622
Elastase	1U4G	−7.752	−5.949	−5.620	−5.663

## Data Availability

The original contributions presented in the study are included in the article.
